# The Reliability of Photograph-Based Digital Measurements for Assessing the Pediatric Elbow Range of Motion—A Pilot Study

**DOI:** 10.3390/children13030336

**Published:** 2026-02-27

**Authors:** Alec C. Smith, Laura L. Bellaire, Joshua N. Speirs, Joel D. Turtle, Bruce A. MacWilliams, Christopher A. Makarewich

**Affiliations:** 1 Department of Orthopaedics, University of Utah, Salt Lake City, UT 84112, USA; 2 Primary Children’s Hospital, Salt Lake City, UT 84113, USA

**Keywords:** pediatric, fracture, elbow, range of motion, telehealth, telemedicine, photograph

## Abstract

**Highlights:**

**What are the main findings?**

- When comparing photograph-based digital measurements to in-office goniometer measurements, accuracy bias (the average difference between observer measurements and the goniometer) showed an overestimation of photographs for extension by 1.2°, an overestimation of photographs for flexion by 5.5°, and an underestimation of photographs for the carrying angle of 3.5°.
- Intra- and inter-rater reliability correlations were all within the good (0.75–0.9) to excellent (>0.9) ranges. The intra-rater intraclass correlation coefficient (ICC) was 0.91 for extension, 0.87 for flexion, and 0.87 for the carrying angle. The inter-rater ICC was 0.98 for extension, 0.93 for flexion, and 0.96 for the carrying angle.

**What are the implications of the main findings?**

- Photograph-based digital measurements appear to be an acceptable alternative compared to in-office goniometer measurements in select pediatric patients.
- The incorporation of this technique in telemedicine evaluations could potentially enhance access to care, reduce the patient and caregiver burden of in-person visits and offer a new avenue to safely streamline upper extremity fracture care in pediatric patient populations.

**Abstract:**

Background/Objectives: The follow-up for pediatric elbow injuries often involves in-office visits to assess the range of motion (ROM). There is evidence for the use of telemedicine and digital assessments of elbow motion in adults but none in pediatrics. The aim of this study was to compare measurements of the joint range of motion and elbow alignment in children, taken in a doctor’s office, with those obtained from photographs and to evaluate the intra- and inter-observer reliability of these measurements. Methods: Fourteen children were prospectively included in this study during follow-ups after an arm fracture. Only the uninjured arm was evaluated: goniometric measurements performed in the office were compared with digital measurements obtained from photographs. For the uninjured arm, in-office goniometer measurements were compared to picture-based digital measurements. Mean digital values were compared to in-office goniometer measurements using a paired *t*-test, and the intra- and inter-rater reliability of the digital measurements was calculated. Results: When comparing photograph-based digital measurements to in-office goniometer measurements, the mean absolute error (average absolute difference between observer measurements and goniometer) showed differences of 4.5° for extension, 7.4° for flexion, and 4.8° for the carrying angle. The accuracy bias (average difference between observer measurements and goniometer) showed an overestimation of photographs for extension by 1.2°, an overestimation of photographs for flexion by 5.5°, and an underestimation of photographs for a carrying angle of 3.5°. Intra- and inter-rater reliability correlations were all within the good (0.75–0.9) to excellent (>0.9) ranges. The intra-rater intraclass correlation coefficient (ICC) was 0.91 for extension, 0.87 for flexion, and 0.87 for the carrying angle. The inter-rater ICC was 0.98 for extension, 0.93 for flexion, and 0.96 for the carrying angle. Conclusions: Photograph-based digital measurements appear to be an acceptable alternative compared to in-office goniometer measurements in pediatric patients.

## 1. Introduction

Elbow fractures are common in children, accounting for approximately 15% of all fractures in pediatric patients [1]. Treatment options vary but often involve immobilization with casts and splints, creating the potential for post-injury stiffness or contracture. The follow-up for these injuries often involves in-office visits to assess the range of motion (ROM) and alignment during the healing and/or remodeling process [2,3]. While important to ensure a good outcome, these in-office visits can be a burden for families, often requiring additional costs and time away from school and work for patients, caregivers, and even siblings [4,5]. Additionally, pediatric orthopedic specialists are concentrated in urban centers, leaving swaths of US patients less than optimally served in rural areas [6].

Telemedicine has emerged as a viable option in orthopedic care, with studies demonstrating a decreased cost and distance traveled, improved family satisfaction, and comparable outcomes compared to in-person clinic visits [7,8,9]. There are also potential drawbacks to this approach, with cited concerns including equal access to care and patient and family perceptions of quality and comparability to in-person visits. Within pediatric orthopedics, in a study of 67 telehealth visits Wong et al. [10] noted that patients were satisfied with initiating the telemedicine visit and felt comfortable discussing needs with the provider and the visit saved time. However, fewer patients felt that telemedicine was of a comparable quality to an in-person visit [10]. Hogue et al. noted that telehealth visits had comparable patient satisfaction to in-person visits; however, telehealth visits had lower ratios of non-White patients, Hispanic patients, and patients with public insurance [11].

Despite the potential limitations, the use of telemedicine to assess the joint ROM remotely is becoming a feasible option for orthopedic providers through digital photography, videography and smartphone/computer-based applications. There is some evidence for the use of these techniques in adults, and several studies have validated the reliability of these methods for the shoulder, elbow and knee [12,13,14,15,16]. Specifically, regarding the elbow ROM, photograph-based goniometry tools have been shown to have high reliability and validity, performing similarly to standard methods such as in-office goniometer measurements [12,15,17].

Children present a unique challenge for using these methods, with potential difficulties in compliance and positioning. To date, no study has fully evaluated the use of photograph-based ROM measurements in pediatric patients. Given this, the primary purpose of this study was to compare the in-office elbow ROM of pediatric patients to those obtained by photographs, with a secondary purpose of assessing the intra- and inter-rater reliability of these measurements.

## 2. Materials and Methods

Over a 9-month period, fourteen pediatric patients (ages 4–11 years) were prospectively included in this pilot study after undergoing operative treatment of a supracondylar humeral fracture using a sample of convenience design (not consecutively included). Children with a prior injury to the currently uninjured arm and those with neurologic impairment and neuromuscular conditions were excluded. Their uninjured arms were studied, and in-office ROM and carrying angle assessments were performed with a universal goniometer at the time of the first post-operative visit. One arm of the goniometer was aligned with the long axis of the upper arm to the center of rotation of the elbow, and the second arm was aligned with the long axis of the forearm. A single in-office goniometer measurement was performed. Families were sent home with written instructions to take standardized digital images of the patient illustrating the flexion, extension and carrying angle of both elbows (Appendix A). The uninjured arm was chosen for study as it would not be affected by post-operative/post-casting stiffness and would be unlikely to change from the time of the first post-operative visit to the ROM visit.

**Figure 1:**
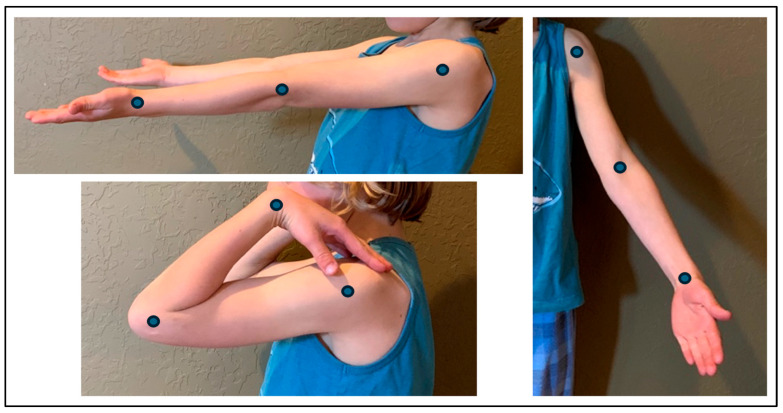
Example photographs showing axis points for photograph-based digital measurement of elbow extension, flexion, and carrying angle.

Elbow photographs were taken at home and sent digitally to the clinic prior to their subsequent ROM telehealth visit. In-office goniometer measurements were compared to picture-based digital measurements. Four pediatric orthopedic surgeons digitally measured each elbow angle for each patient twice, at least seven days apart, using Adobe Photoshop, for a total of 336 unique measurements. Measurements were made along the long axis of the upper arm and forearm, connecting central points from the shoulder, elbow, and wrist (Figure 1) following previously reported techniques [12,17]. Photograph-based measurements were performed in a blinded fashion (each observer was blinded to the other observers’ measurements, and each of the two individual observer’s measurements were blinded from themselves and separated by at least 7 days).

Statistical analyses compared the digital to in-office goniometer measurements and the mean absolute error (average absolute difference between observer measurements and goniometer) with paired *t*-tests. The accuracy bias (average difference between observer measurements and goniometer) and intra- and inter-rater reliability of the digital measurements were also calculated. The intraclass correlation coefficient (ICC) was used (intra-rater ICC (2,1)—model = “two-way”, type = “agreement”, unit = “single”; inter-rater ICC (2,k)—model = “two-way”, type = “agreement”, unit = “average”). These were classified as poor < 0.50, moderate 0.50–0.75, good 0.75–0.90, and excellent > 0.90 [18]. Systematic differences between observer image measurements and the corresponding goniometric result were assessed to evaluate measurement accuracy and bias. The normality of the paired differences for each measure (extension, flexion, and carrying angle) was formally evaluated using the Shapiro–Wilk test. As data were normal, paired *t*-tests adjusted using the Holm procedure for multiple comparisons were employed to determine if the mean error significantly deviated from zero, indicating a systematic over- or underestimation. Statistical significance was defined at an alpha level of 0.05, and all analyses were conducted in R. There were no missing data.

## 3. Results

Among the 14 patients included, the average age was 6 years (range 4–11 years), and the median age was 6 years. There were eight males and six females with 11 Right and 3 Left elbows. The average time between the in-office measurement and home photographs was 85 days (range of 55–139 days). In-office goniometer measurements showed an elbow extension range from 0°/neutral to 15° of hyperextension. The range of flexion was 130° to 145°. The carrying angle ranged from 4° to 15° of valgus. Photograph-based digital measurements showed an elbow extension range from 9.4° of flexion to 20.5° of hyperextension. The range of flexion was 133° to 152°, and the carrying angle ranged from 8.7° to 14.2° of valgus.

When comparing the photograph-based digital measurements to in-office goniometer measurements, the mean absolute error showed absolute differences of 4.5° for extension (*p* = 0.043), 7.4° for flexion (*p* < 0.001), and 4.8° for the carrying angle (*p* < 0.001). The accuracy bias showed an overestimation of photographs for extension by 1.2°, an overestimation of photographs for flexion by 5.5°, and an underestimation of photographs for the carrying angle of 3.5°. Intra- and inter-rater reliability correlations were all within the good (0.75–0.9) to excellent (>0.9) ranges. The intra-rater ICC was 0.91 for extension, 0.87 for flexion, and 0.87 for the carrying angle. The inter-rater ICC was 0.98 for extension, 0.93 for flexion, and 0.96 for the carrying angle. Table 1 shows the error and absolute error for the photograph-based digital measurements for each angle by individual readers. Table 2 shows the ICC values and confidence intervals.

## 4. Discussion

The results of this study demonstrate that photograph-based digital elbow ROM and carrying angle measurements showed good to excellent intra- and inter-rater reliability, while demonstrating a strong agreement with in-office goniometer measurements. The standard error of goniometer measurements in the elbow has been noted to be upwards of 10°. In a study of 42 individuals, Cleffken et al. identified the smallest detectable difference for flexion/extension to range from 4.2 to 10.8° depending on the upper arm position [19]. Armstrong et al. noted a meaningful change in intra-tester ROM measurements with a 95% confidence as being greater than 6° for flexion and 7° for extension in a study of 38 patients [20]. The inter-tester meaningful change was found to be 10° for both flexion and extension. Chapleu et al., in a study of 51 subjects, identified that the maximal errors of goniometer measurements were 10.3° for extension, 7.0° for flexion, and 6.5° for carrying angles 95% of the time [21]. In our study, the mean absolute errors of photograph-based measurements for the extension, flexion, and carrying angle were statistically significantly different when considering in-office vs. photograph-based techniques; however, the mean absolute error and accuracy bias were all within or below these previously reported ranges. Our study found a mean absolute error range of 4.5–7.4° and an accuracy bias of 1.2–5.5° for photograph-based measurements, with these falling within or below the range of previously reported goniometer measurement errors of 4.2–10.8° from other studies, as reported above. This highlights that photograph-based digital measurements appear to be an acceptable alternative to in-office goniometer measurements in pediatric patients.

The prior literature in adult populations has shown a similar reliability to our study when validating digital ROM measurements of the elbow. Blonna et al. evaluated the reliability of photograph-based goniometry measurements of the elbow in 50 participants among four observers of varying levels of expertise [12]. Their study demonstrated an excellent intra-observer reliability ranging between 0.93 and 0.99, while an inter-observer reliability of 0.89–0.98 was also considered excellent. Systematic errors ranged between −3 to 2° and 0 to +/−4°, respectively. Another study conducted by Meislin et al. examined 32 participants (64 elbows) with a similar methodology to digitally assess the elbow ROM [15]. Photograph-based ROM measurements correlated highly with goniometer-based methods and displayed no statistically significant difference between measurement methods. Furthermore, Keijsers et al. conducted a 40-patient study assessing digital elbow ROM measurements using digital photographs, movies, and a goniometry smartphone application. They found a good to excellent intra- and inter-observer reliability, alongside a good to excellent correlation with in-person goniometer measurements of flexion and extension angles [13]. These findings are consistent with our results, with the intra-rater reliability in the good to excellent range (intra-rater ICC 0.87–0.91 and inter-rater ICC 0.93–0.98).

The use of telemedicine in orthopedics has been growing, with recent studies demonstrating that it is possible to provide the same level of care to patients as in-office visits while decreasing costs and improving convenience. In a series of two studies, Buvik et al. reviewed their experience using telemedicine for orthopedic consultations in Norway [4,22]. They found that patients evaluated by remote video visits had similar satisfaction and health scores to those seen in person. Furthermore, a high proportion of patients selected a remote telemedicine encounter for their next visit. In addition, these clinics provided a large cost savings both for the patients and the health system. Specifically in pediatric fracture care, McGill et al. described their experience using telemedicine to deliver outreach fracture clinics in Australia. They found that telemedicine improved access to care and led to substantial cost savings [5]. Sinha et al. evaluated patient/family satisfaction with this technique, comparing 66 patients treated in a conventional setting and 101 patients treated via telemedicine [9]. They found a similar satisfaction between groups, with significantly decreased costs and travel times in the telemedicine group. In addition, Silva et al. performed a randomized controlled trial for nondisplaced pediatric elbow fractures, with a total of 52 patients randomized to in-person vs. telemedicine follow-ups for their fourth-week follow-up appointment [8]. They found no difference in the fracture displacement, range of motion, or pain scores between groups and higher satisfaction in the patients seen via telemedicine. One study by Magno et al. does evaluate the use of these techniques in pediatric patients [14]. The authors compare in-office elbow range of motion goniometer measurements with photographs measured on a cell phone-based app, with images obtained by the physicians during the same visit. They found no statistically significant difference between measurement modalities. While this does provide support for the use of photograph-based measurements, as the images were obtained by the physicians in a controlled and standardized fashion, it may limit the application to telehealth/remote follow-ups in which families would be obtaining the images. The ability to include an accurate assessment of the ROM through telehealth can potentially help expand the use of this approach in pediatric fracture care.

There are limitations to this study. The sample size is relatively small, which necessarily limits the generalizability of the results. Findings describe measurements of uninjured elbows without pre-existing deformities or contractures. Both in-person and photograph-based digital measurements are likely to prove more challenging in patients with multiplanar deformities or significant soft tissue swelling. Pediatric patients with underlying behavioral or developmental challenges may be less cooperative with and amenable to photograph-based measurements. Additionally, this study evaluates the uninjured arm, and the utility of this technique in an injured arm requires additional study. While the results are promising, this study would benefit from a larger cohort to obtain a more complete picture of measurement variances across diverse populations. Further studies could also attempt to correlate patient factors such as age, the time from the initial measurement, etc., with better ICCs. Additionally, there was variability in the quality of patient-submitted photographs. Instructions were provided to families in hopes of standardizing images, but there were clear differences in the distance and angle of the camera relative to the arm between patients. There were also subtle but visible differences in arm rotation, with potential to skew angle measurements. The same is possible with in-person measurements with a goniometer, but when assessing photographs, patient repositioning is not possible.

Patient positioning was expected to generate unique challenges in this pediatric population. However, the photograph-based digital measurements were well within the standard error of goniometer measurements. Of note, the photo quality, lighting and patient clothing, such as shirts, may also play roles in the ability to accurately measure the elbow ROM with photograph-based digital measurements. Refined instructions and possibly the use of video or live photo capture by telehealth, in which patients and families could be directed in real time, could potentially improve the accuracy of the measurements.

## 5. Conclusions

Photograph-based digital measurements appear to be an acceptable alternative compared to in-office goniometer measurements in select pediatric patients. The incorporation of this technique in telemedicine evaluations could potentially enhance access to care, reduce the patient and caregiver burden related to in-person visits and offer a new avenue to safely streamline upper extremity fracture care in pediatric patient populations. Further larger studies will be needed to confirm the use of this technique in injured arms and the application to other patient populations.

## Figures and Tables

**Table 1:** Error and absolute error for the photograph-based digital measurements for each angle by individual reader.

	Error (Mean ± SD)				Absolute Error (Mean ± SD)			
	Reader 1	Reader 2	Reader 3	Reader 4	Reader 1	Reader 2	Reader 3	Reader 4
Carrying Angle	3.7 (4.5)	3.5 (5.1)	4.4 (5.0)	2.6 (5.0)	4.7 (3.5)	4.7 (4.0)	5.4 (3.9)	4.3 (3.5)
Extension	−3.0 (6.3)	−0.5 (6.4)	−0.5 (6.2)	−0.8 (5.6)	5.2 (4.5)	4.3 (4.7)	4.3 (4.5)	4.3 (3.6)
Flexion	−4.8 (6.8)	−6.9 (6.0)	−3.4 (6.1)	−6.8 (6.5)	7.3 (3.9)	7.9 (4.5)	6.1 (3.2)	8.3 (4.3)

**Table 2:** Intra- and Inter-rater reliability findings.

Metric	Intra- Mean	Intra 5th %	Intra 95th %	Inter- Mean	Inter 5th %	Inter 95th %
Carrying Angle	0.867	0.645	0.955	0.959	0.904	0.986
Extension	0.908	0.757	0.968	0.975	0.941	0.991
Flexion	0.871	0.667	0.956	0.925	0.698	0.978

**Figure A1:**
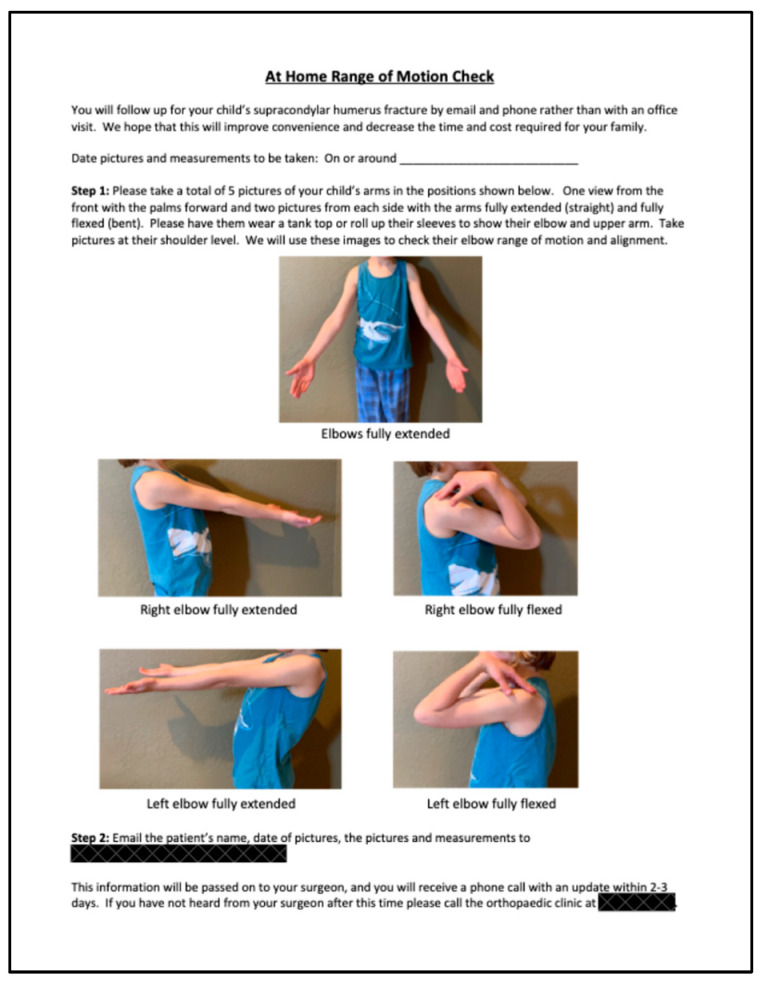
Figure Showing Instructions Given to Families to Obtain Elbow Range of Motion Photographs at Home.

## Data Availability

The raw data supporting the conclusions of this article will be made available by the authors on request.
